# ABA Speeds Up the Progress of Color in Developing *F. chiloensis* Fruit through the Activation of *PAL*, *CHS* and *ANS*, Key Genes of the Phenylpropanoid/Flavonoid and Anthocyanin Pathways

**DOI:** 10.3390/ijms23073854

**Published:** 2022-03-31

**Authors:** Elena Mattus-Araya, Joselin Guajardo, Raúl Herrera, María A. Moya-León

**Affiliations:** Laboratorio de Fisiología Vegetal y Genética Molecular, Instituto de Ciencias Biológicas, Universidad de Talca, Talca 3465548, Chile; elena.mattus@utalca.cl (E.M.-A.); joguajardo@utalca.cl (J.G.); raherre@utalca.cl (R.H.)

**Keywords:** ABA, anthocyanins, anthocyanins pathway, antioxidants, color, flavonoid pathway, phenolics, phenylpropanoid pathway, qPCR, Fluridon

## Abstract

Phenolic compounds with antioxidant properties have risen in interest due to their benefits for human health. *Fragaria chiloensis* is a native wild berry species from Chile that develops a white/pink receptacle and white flesh at the ripe stage. Changes in color parameters, anthocyanins, secondary metabolites (phenolics, flavonoids), and total antioxidant capacity were followed during the development and ripening of *F. chiloensis* fruit. The increment in color ‘a’ index takes place in parallel with anthocyanins rise and the reduction in phenolics, flavonoids, and antioxidant capacity. Good correlations were determined between color development, anthocyanins, and the expression of key phenylpropanoid/flavonoid and anthocyanin pathway genes. To investigate the role of ABA on color development, detached immature fruit (C2 stage) were treated with exogenous ABA and stored at 20 °C. Fruit color development was accelerated by ABA treatment compared to non-treated fruit, and consistent with that, the increment in the accumulation of anthocyanins and transcripts of phenylpropanoid/flavonoid, and anthocyanin pathways genes such as *FcPAL*, *FcCHS*, and *FcANS* were observed. This suggests that ABA promotes transcriptional changes that lead to the color formation on this non-climacteric fruit.

## 1. Introduction

Nowadays, humans are interested in healthy diets and berries are an excellent source of polyphenolics with antioxidant properties which may protect against oxidative-stress conditions related to illness [1]. *Fragaria chiloensis* (L.) Mill. is a native wild berry species from Chile, dispersed along the central-south part of Chile between the Andes and the coastal mountains [2]. Two botanical forms have been identified in *F. chiloensis* (L.) Mill. subsp. chiloensis based on characteristics such as plant size, color, and fruit size: the ‘forma patagonica’ with small red fruit and the ‘forma chiloensis’ with large fruit size, white/pink receptacle, and white flesh [3,4]. The white Chilean strawberry (*F. chiloensis* (L.) Mill. subsp. chiloensis *f. chiloensis*) has been cultivated by pre-Columbian populations, and through traditional selection methods, plants producing big fruits of white/pink color have been selected [2]. This non-climacteric fruit is appreciated in the market for its excellent organoleptic attributes such as taste and aroma, in addition to its exotic white-pink color. In the local market, its commercialization price is at least ten times higher than commercial strawberry *(F. x ananassa*). In addition, it has been reported a high antioxidant activity for the fruit [5,6], and health benefits of its consumption [7,8,9], supporting the fruit as a functional food. Nevertheless, like many berries, the fruit has a short shelf life mainly due to its fast and intense softening [10]. 

This fruit offers an interesting model to study fruit pigmentation, considering the white/pink color of its receptacle and the white flesh. The typical red color of strawberry fruit is due to anthocyanins, with a major abundance of pelargonidin 3-glucoside; however, the Chilean strawberry fruit developed a white-pink color due to a low level of anthocyanins; the main compound quantified is cyanidin 3-glucoside, followed by pelargonidin 3-glucoside in lesser proportions [6]. Anthocyanins are synthesized through the phenylpropanoid and flavonoid biosynthesis pathways, metabolic pathways that convert phenylalanine into anthocyanins, flavonols, flavons, flavan-3-ols, and proanthocyanidins. Intermediates and final compounds of the route also contribute to the antioxidant machinery of the fruit.

The complete transcriptional profile of genes involved in flavonoid biosynthesis has been described during the development of the *F. chiloensis* fruit [11]. The expression of structural genes of this complex pathway is highly regulated. Several transcription factors (TF) regulate the flavonoid biosynthesis pathway, especially MYB family proteins. *FaMYB1*, a TF isolated from *Fragaria × ananassa*, was reported to suppress the accumulation of certain flavonoid compounds in tobacco flowers [12]. Moreover, the silencing of *FaMYB1* by RNA interference resulted in a significant increase in anthocyanin content [13]. *F. chiloensis MYB1* ortholog *(FcMYB1)* was identified and belongs to the R2R3-MYB repressor group [14]. The expression profile of *FcMYB1* slightly increased during the ripening of *F. chiloensis* fruit, showing higher levels of transcripts than *Fragaria × ananassa.* It was hypothesized that the higher level of *FcMYB1* transcripts could contribute to the pigment-deficient phenotype of the fruit. Then, *FcMYB1*-suppressed fruit showed an increased accumulation of pelargonidin 3-glucoside, in addition to alterations in the transcription level of genes of the flavonoid biosynthesis pathway such as down-regulation of *CHI*, *F3H*, *DFR*, *LAR*, and *ANR*, up-regulation of *ANS* and *UFGT*, and no change in *CHS*, confirming the pleiotropic regulation effect of *FcMYB1* [14].

Hormonal regulation studies on color formation have been mainly investigated in *F. x ananassa* fruit. Jia et al. (2011) [15] proved that silencing *FaNCED1*, an ABA biosynthesis gene, resulted in decreased levels of ABA and non-colored fruit. Moreover, the addition of exogenous ABA rescued the uncolored phenotype. In a recent study, it was proven that *FaMYB1* and *FaMYB10* reverberate directly in anthocyanin accumulation in response to the elevations of endogenous ABA concentration [16].

In order to improve our knowledge of this native exotic white-pink species, the effect of ABA on color development was evaluated. For that, developing fruit (large green stage) was subjected to ABA treatments following changes in anthocyanins and the transcription level of phenylpropanoid and flavonoid biosynthesis pathway genes. The effect of Fluridon treatment, an inhibitor of ABA biosynthesis, was also assayed. Changes in the content of important metabolites of the pathway related to functional food properties, such as flavonoids and polyphenols, and their antioxidant capacity were also analyzed.

## 2. Results

### 2.1. Development of Color Changes and Secondary Metabolites during Development of F. chiloensis Fruit

**Figure 1:**
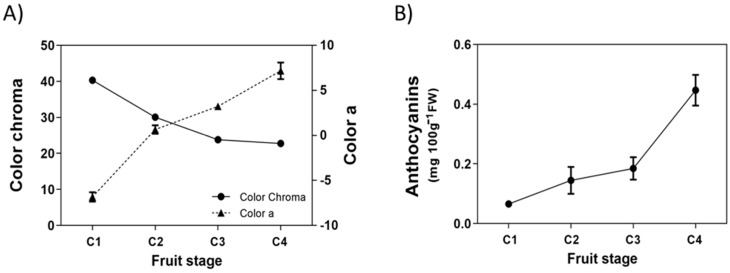
**Changes in fruit color during development of *Fragaria chiloensis* fruit**. (**A**) Changes in color determined by the colorimeter and expressed as chroma and ‘a’ value; (**B**) changes in anthocyanins content. Values correspond to the mean ± SE of three replicates of five fruit each.

Even though the white Chilean strawberry does not develop an intense red color in its receptacle, there are some changes in the general appearance of the fruit during its development and ripening. The color of its receptacle changes from green at the immature stage to white/pink color at the ripe stage, meanwhile the color of achenes changes from green to red. These changes were detected by the colorimeter, as chroma values decrease during fruit development which reflects the disappearance of the green background from receptacles and achenes (Figure 1A). These changes are more evident when the parameter ‘a’ is evaluated; the C1 stage has a negative ‘a’ value (green color) that becomes positive (red color) and greater in magnitude as the fruit develops and ripens (Figure 1A). In parallel with this, there is a small increment in the level of anthocyanins during ripening, and the maximum level reached in this fruit lot was 0.45 mg per 100 g FW (Figure 1B). 

**Figure 2:**
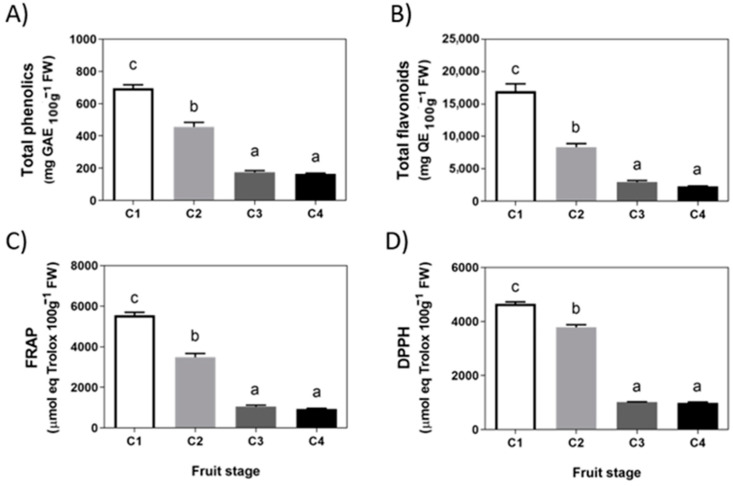
**Changes in phenolics and antioxidants during development of *Fragaria chiloensis* fruit.** (**A**) Changes in phenolic compounds content; (**B**) level of flavonoids content; (**C**) changes in antioxidant content determined by FRAP method; (**D**) changes in antioxidant content determined by DPPH method. Values correspond to the mean ± SE of three independent extractions and determinations performed in triplicates. Different letters indicate significant differences between fruit stages. One-way ANOVA with Dunnett correction post hoc was used.

A series of metabolites related to the phenylpropanoids pathway were evaluated in *F. chiloensis* fruit. The total content of phenolics and flavonoids decreases significantly between stages C1 and C3 and remains at a low level until the end of *F. chiloensis* ripening (Figure 2A,B). Levels of 166 mg GAE per 100 g FW for phenolics and 2273 mg QE per 100 g FW for flavonoids were determined in fruit at the ripe stage. A similar pattern of decrease occurs with the antioxidant capacity determined as FRAP and DPPH (Figure 2C,D). At the ripe stage, *F. chiloensis* fruit ends with FRAP and DPPH values of 935 and 994 μmol Trolox equivalents per 100 g FW, respectively.

### 2.2. Changes in Response to ABA Treatment

**Figure 3:**
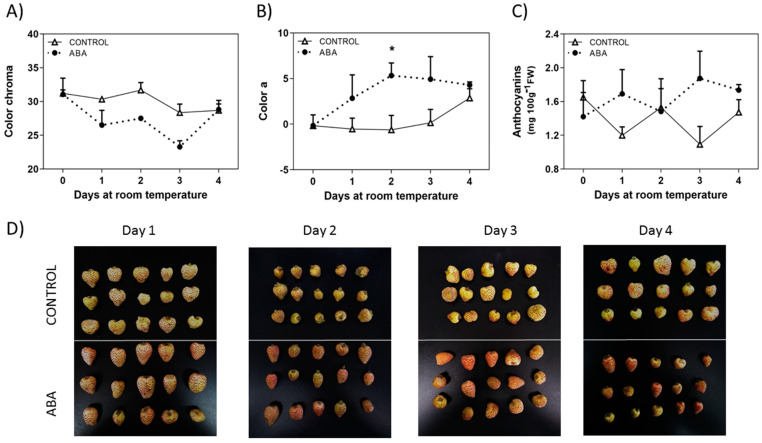
**Changes in fruit color induced by ABA treatment.** The pedicel of *F. chiloensis* fruit at the C2 stage was submerged in 1 mM ABA or only buffer; the fruit was maintained at room temperature. (**A**) Changes in color expressed as chroma; (**B**) changes in color expressed as a* (green to red, SCIELAB scale); (**C**) changes in anthocyanins content. Values correspond to the mean + SE of three replicates of five fruit. Asterisks indicate significant differences between treatments (* *p* < 0.05; disappeared *t*-test with Bonferroni correction post hoc). (**D**) Images reporting the changes in color in the fruit treated with ABA or non-treated. Photos were taken after 1, 2, 3, and 4 days of treatment.

It has been recently reported the effect of ABA treatment on softening of *F. chiloensis* fruit [17]. In addition to firmness reduction, ABA also accelerates changes in color development of *F. chiloensis* fruit as indicated by the faster reduction in chroma values and the quicker increment in the ‘a’ parameter observed in ABA treated fruit compared to non-treated fruit (Figure 3A,B). In other words, fruit at the C2 stage with low levels of ABA [17] treated with exogenous ABA took 2 days to reach the final red color (‘a’ parameter) compared to 4 days spent by non-treated fruit to reach the same final color (Figure 3B). The changes in the anthocyanins content are not as clear as direct color determinations; however, there is a notorious increment in anthocyanins in ABA-treated fruit compared to non-treated fruit (Figure 3C), although changes are not statistically significant. Photographs of the fruit taken during the experiment confirm the changes in color development (Figure 3D).

**Figure 4:**
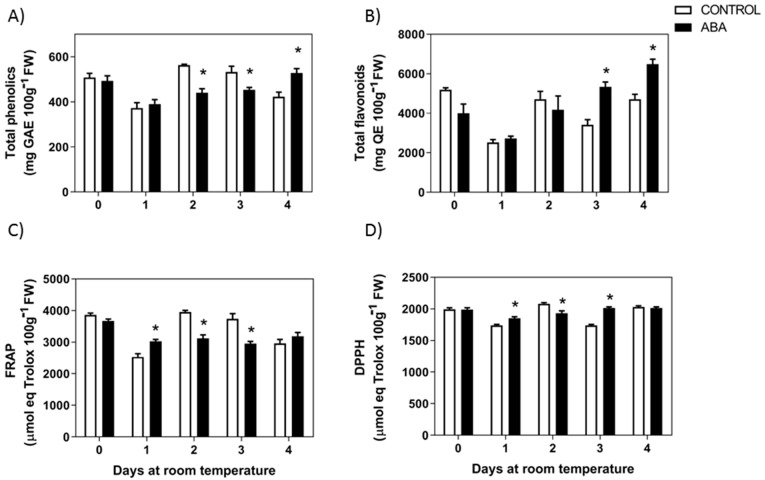
**Changes in phenolics and antioxidants in response to ABA treatment.** The pedicel of *F. chiloensis* fruit at the C2 stage was submerged in 1 mM ABA or only buffer, and maintained at room temperature. (**A**) Changes in phenolic compounds content; (**B**) level of flavonoids content; (**C**) changes in antioxidant content determined by FRAP method; (**D**) changes in antioxidant content determined by DPPH method. Values correspond to the mean ± SE of three independent extractions and determinations performed in triplicates. Asterisks indicate significant differences between treatments (* *p* < 0.05). Two-way ANOVA with Tukey correction post hoc was used.

When *F. chiloensis* fruit at the C2 stage was treated with ABA, there was also a reduction in the level of total phenolics after 2 or 3 days of treatment (Figure 4A). On the other hand, the content of flavonoids temporarily decreases during the first two days in response to ABA but then increases significantly compared to control fruit and fruit from day 0 (Figure 4B). Significant changes in the antioxidant capacity were also observed in response to ABA; a significant reduction in FRAP and DPPH levels were observed after 1, 2, and 3 days of treatment (Figure 4C,D). These indicate that ABA accelerates the reduction in total phenolics and antioxidants in *F. chiloensis* fruit. Interestingly, there is a general increment in anthocyanins and total flavonoids content in response to ABA.

### 2.3. Expression Level of Anthocyanin Biosynthetic Pathway Genes during Fruit Development and in Response to ABA Treatment

In order to understand the appearance of color in strawberry fruit, the expression level of genes that drives the biosynthesis towards anthocyanins was followed during fruit development. The entire biosynthetic pathway can be split into different metabolic pathways. PAL (phenylalanine ammonium lyase), C4H (cinnamate 4-hydroxylase), and 4CL (4-coumarate:CoA ligase) corresponds to the first three reactions of the phenylpropanoid biosynthesis pathway that transforms phenylalanine into 4-coumaroyl-CoA. The following reactions belong to the flavonoid biosynthesis pathway and involve those performed by CHS (chalcone synthase) and CHI (chalcone isomerase) that transforms 4-coumaroyl-CoA and malonyl-CoA into the flavanone naringenin. Then F3H (flavanone 3β-hydroxylase) converts naringenin into the dihydroflavonol dihydrokaempferol, which is then transformed into dihydroquercetin by F3′H (flavonoid 3′-hydroxylase). At this point, the pathway can be diversified towards flavonols by means of FLS (flavonol synthase) or towards anthocyanins by DFR, ANS, and UFGT. DFR (dihydroflavonol 4-reductase) promotes the synthesis of leucoanthocyanidins, ANS (anthocyanidin synthase) the synthesis of anthocyanidins, and UFGT (UDP-glucose:flavonoid 3-O-glucosyltransferase) the synthesis of anthocyanins. Leucoanthocyanidins can also be transformed into flavan-3-ols by means of LAR (leucoanthocyanidin reductase), and anthocyanidins can be transformed into epi-flavan-3-ols by means of ANR (anthocyanidin reductase). Flavan-3-ols and epi-flavan-3-ols can then be converted into proanthocyanidins.

**Figure 5:**
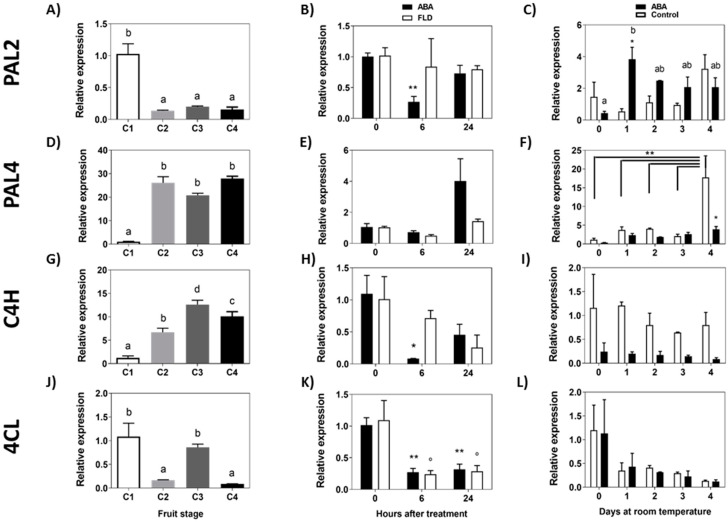
Expression level of phenylpropanoid pathway genes during development of *F. chiloensis* fruit and in response to ABA treatment. In the short-term treatment, fruit at C2 stage was immersed in 1 mM ABA or 100 µM Fluridon (FLD). In the long-term treatment the pedicel of the fruit submerged in 1 mM ABA or only buffer. Changes in the expression level of each gene during fruit development are shown on the left-hand side of the figure; in the center the effect of ABA/Fluridon in the short-term treatment; on the right-hand side the effect of ABA in the long-term treatment. The expression level of genes from the first portion of the phenylpropanoid pathway was determined through qPCR analysis: *FcPAL2*, phenylalanine ammonia-lyase 2 (**A**–**C**); *FcPAL4*, phenylalanine ammonia-lyase 4 (**D**–**F**); *FcC4H*, trans-cinnamate 4-hydroxylase 1 (**G**–**I**); *Fc4CL*, 4-coumarate-CoA ligase (**J**–**L**). In expression analysis during development progress of *F. chiloensis,* different letters indicate significant differences between fruit stages (one-way ANOVA with Dunnett correction post hoc). In ABA short-term treatments, asterisks or circles indicate significant differences compared to time 0 for each hormone/inhibitor treatment (* *p* < 0.05, ** *p* < 0.01; one-way ANOVA with Dunnett correction post hoc). For ABA long-term treatment, asterisks indicate significant differences between treatments (* *p* < 0.05, ** *p* < 0.01), different letters indicate significant differences between days of ABA treatment, and bars with asterisks indicate significant difference between days of control group; two-way ANOVA with Tukey correction post hoc test was used.

Two *PAL* genes have been identified so far in *F. chiloensis*, *FcPAL2* and *FcPAL4*. The accumulation of *FcPAL2* transcripts decreases early during the development of the fruit, with sharp reductions during the transition from the C1 to C2 stage, maintaining low expression levels from the C2 stage until the end of ripening (Figure 5A). Meanwhile, *FcPAL4* transcripts increase intensively during the transition from the C1 to C2 stage, maintaining a high expression level from the C2 to C4 stage (Figure 5D). In response to ABA, there is a significant reduction in the accumulation of *FcPAL2* transcripts at 6 h, and no changes in expression were observed in Fluridon-treated fruit (Figure 5B). In the longer time, there is a significant increment in *FcPAL2* transcripts in response to ABA during the following 4 days of storage (Figure 5C). In the case of *FcPAL4*, a non-significant increase in transcripts after 24 h could be observed (Figure 5E) and no changes in response to Fluridon in the short-term experiment. When the effect was tested during a longer time frame, a small increment in transcript accumulation of *FcPAL4* was observed in ABA treated fruit after 4 days of storage, and the same increment was observed in control fruit (Figure 5F). 

The accumulation of *FcC4H* transcripts increases early during the development of the fruit, with the maximum level at the C3 stage (Figure 5G). In response to ABA, there is a notorious decrease in *FcC4H* transcripts after 6 h of treatment which recovers at 24 h; meanwhile, in Fluridon-treated fruit, transcript levels remain almost unchanged (Figure 5H). When the effect of ABA was tested during a longer period of time, a non-significant change in transcripts for *FcC4H* was determined in ABA-treated fruit or control fruit (Figure 5I). *Fc4CL* displays an oscillating transcription profile during fruit development; the level of transcripts decreases during the transition between C1 and C2 stages, increases between C2 and C3, and decreases again between C3 and C4 stages (Figure 5J). In response to ABA and Fluridon, there is a significant decrease in *Fc4CL* transcript levels (Figure 5K). In the longer time, non-significant differences were observed in ABA-treated fruit or the control group (Figure 5L). In summary, considering the phenylpropanoid biosynthetic pathway, the treatment of ABA in the longer time induces the expression of the first gene of the route, *FcPAL2* and *FcPAL4*. In the short time, ABA transiently represses the expression of *FcPAL2*, and interestingly Fluridon avoids this transcriptional change. 

**Figure 6:**
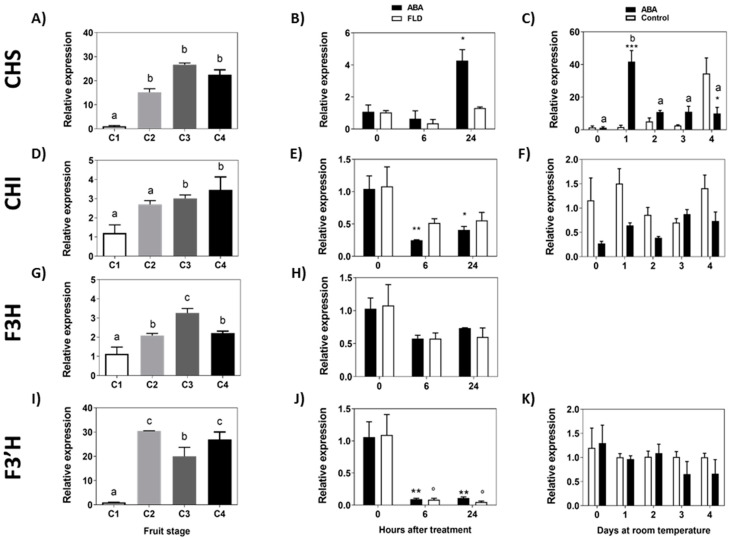
Expression level of the genes from the initial portion of the flavonoid pathway during development of *F. chiloensis* fruit and in response to ABA treatment. Fruit at C2 stage was immersed in 1 mM ABA or 100 µM FLD (short-term treatment) or fruit pedicel was submerged in 1 mM ABA or only buffer (longer-time treatment). Changes in the expression level of each gene during fruit development are shown on the left-hand side of the figure; in the center the effect of ABA/FLD in the short term-treatment; on the right-hand side the effect of ABA in the long-term treatment. Gene expression levels of the flavonoid pathway were quantified through qPCR analysis: *FcCHS*, chalcone synthase (**A**–**C**); *FcCHI*, chalcone isomerase (**D**–**F**); *FcF3H*, flavanone 3-hydroxylase (**G**,**H**); *FcF3′H*, flavonoid-3′-hydroxylase (**I**–**K**). In expression analysis during development progress of *F. chiloensis,* different letters indicate significant differences between fruit stages (one-way ANOVA with Dunnett correction post hoc). In ABA short-term treatments, asterisks or circles indicate significant differences compared to time 0 for each hormone/inhibitor treatment (* *p* < 0.05, ** *p* < 0.01; one-way ANOVA with Dunnett correction post hoc). For ABA long-term treatment, asterisks indicate significant differences between treatments (* *p* < 0.05, ** *p* < 0.01, *** *p* < 0.001), different letters indicate significant differences between days of ABA treatment; two-way ANOVA with Tukey correction post hoc test was used.

The accumulation of *FcCHS* transcripts increases during the development of the fruit, with a low expression level at the C1 stage and a high expression level between C2 and C4 stages (Figure 6A). In response to ABA, there was a significant increment in the accumulation of *FcCHS* transcripts after 24 h of treatment, and interestingly this increment was avoided by Fluridon (Figure 6B,C). The increment in *FcCHS* expression promoted by ABA is only transient as it was not maintained during the following days (Figure 6C). *FcCHI* showed an increasing expression profile during the development of *F. chiloensis* fruit (Figure 6D). In response to ABA, there is a significant reduction in the accumulation of *FcCHI* transcripts after 6 h and 24 h (Figure 6E); non-significant changes were observed in response to Fluridon. When the effect was tested during a longer period, no changes in the transcript accumulation of *FcCHI* were observed in ABA-treated fruit (Figure 6F). 

Transcripts for *FcF3H* showed an incremental pattern during the development of *F. chiloensis* fruit with maximum expression values at stage C3 (Figure 6G). In response to ABA or Fluridon, there is a non-significant change in *FcF3H* transcript levels (Figure 6H). No expression of *FcF3H* was recorded when ABA was tested in the longer time experiment. Following the pathway, the accumulation of *FcF3′H* transcripts increases sharply between C1 and C2 stages and remains high during the development of the fruit with higher values at C2 and C4 stages (Figure 6I). In response to ABA and Fluridon, there is a notorious decrease in *FcF3′H* transcripts after 6 h and 24 h (Figure 6J); however, *FcF3′H* transcripts remained unchanged in response to ABA during the longer period of time experiment (Figure 6K). In summary, considering the first portion of the flavonoid biosynthetic pathway, the treatment of ABA promotes the temporal induction of *FcCHS* and the transient repression of *FcCHI* and *FcF3′H*. Meanwhile, the transcriptional changes observed for *FcCHS* and *FcCHI* were not detected in Fluridon-treated fruit.

**Figure 7:**
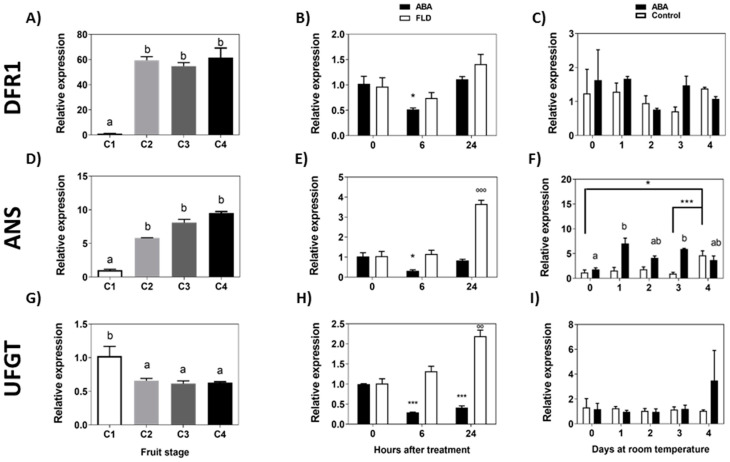
Expression level of the genes from the final portion of the flavonoid pathway and anthocyanin pathway during development of *F. chiloensis* fruit and in response to ABA treatment. Fruit at C2 stage was immersed in 1 mM ABA or 100 µM Fluridon (short-term treatment) or fruit pedicel was submerged in 1 mM ABA or only buffer (longer-time treatment). Changes in the expression level of each gene during fruit development are shown on the left-hand side of the figure; in the center the effect of ABA/FLD in the short-term treatment; on the right-hand side the effect of ABA in the long-term treatment. The expression level of genes of the flavonoid pathway was determined through qPCR analysis: *FcDFR1*, Dihydroflavonol 4-reductase 1 (**A**–**C**); *FcANS*, Anthocyanidin synthase (**D**–**F**); *FcUFGT*, UDP-glucose flavonoid 3-O glucosyltransferase (**G**–**I**). In expression analysis during development progress of *F. chiloensis,* different letters indicate significant differences between fruit stages (one-way ANOVA with Dunnett correction post hoc). In ABA short-term treatments, asterisks or circles indicate significant differences compared to time 0 for each hormone/inhibitor treatment (* *p* < 0.05, *** *p* < 0.001; one-way ANOVA with Dunnett correction post hoc). For ABA long-term treatment, asterisks indicate significant differences between treatments (* *p* < 0.05, *** *p* < 0.001), different letters indicate significant differences between days of ABA treatment, and bars with asterisks indicate significant difference between days of control group; two-way ANOVA with Tukey correction post hoc test was used.

Following the synthesis pathway that ends in anthocyanins, the accumulation of transcripts for *FcDFR1* increases sharply during the transition from the C1 to C2 stage, keeping a high expression level from C2 until the end of ripening (Figure 7A). ABA treatment significantly reduces the expression of *FcDFR1* during the first 6 h but recovers the initial expression level after 24 h; meanwhile, Fluridon does not affect the *FcDFR1* transcript level (Figure 7B). During a longer period of time, the transcript accumulation of *FcDFR1* displays a tendency to diminish with time in response to ABA, although no significant differences were observed compared to control fruit (Figure 7C). 

The accumulation of *FcANS* transcripts increases during the development of the fruit (Figure 7D). ABA treatment transiently reduces the level of transcripts after 6 h that recovers after 24 h; meanwhile, Fluridon induces the level of transcripts after 24 h (Figure 7E). The expression of *FcANS* increments in a significant manner from day 1 to day 4 of treatment in response to a long exposure to ABA (Figure 7F). 

The level of *FcUFGT* transcripts decreases throughout the development and ripening of *F. chiloensis* fruit (Figure 7G). A significant reduction in expression is observed between C1 and C2 stages, and expression remains at a low level until the end of ripening. In response to ABA treatment, the *FcUFGT* transcripts level decreases significantly after 6 h and 24 h of treatment; meanwhile, Fluridon induces the accumulation of transcripts after 24 h (Figure 7H). In the longer time, no changes in *FcUFGT* transcripts were observed in ABA-treated fruit (Figure 7I). In conclusion, in response to ABA, there is a temporal reduction in *FcANS* during the first hours of treatment followed by a significant increment during the following days. These changes were also accompanied by the temporal reduction in the expression of *FcDFR1* and *FcUFGT*. An opposite expression pattern determined in ABA-treated fruit was noticed in response to Fluridon in the case of *FcANS* and *FcUFGT*.

**Figure 8:**
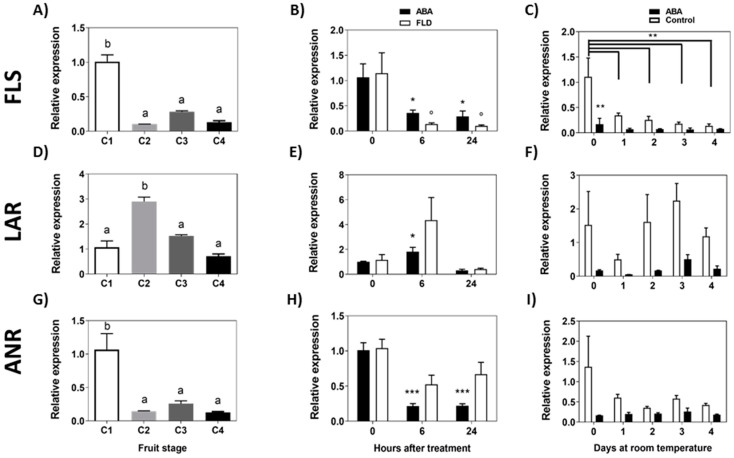
Expression level of genes converting intermediates of the flavonoid pathway during development of *F. chiloensis* fruit and in response to ABA treatment. Fruit at C2 stage was immersed in 1 mM ABA or 100 µM Fluridon (short-term treatment) or fruit pedicel was submerged in 1 mM ABA or only buffer (longer-time treatment). Changes in the expression level of each gene during fruit development are shown on the left-hand side of the figure; in the center the effect of ABA/FLD in the short-term treatment; on the right-hand side the effect of ABA in the long-term treatment. The expression level of genes of the flavonoid pathway was determined through qPCR analysis: *FcFLS*, flavonol synthase (**A**–**C**); *FcLAR*, Leucoanthocyanidin reductase (**D**–**F**); *FcANR*, anthocyanidin reductase (**G**–**I**). In expression analysis during development progress of *F. chiloensis,* different letters indicate significant differences between fruit stages (one-way ANOVA with Dunnett correction post hoc). In ABA short-term treatments, asterisks or circles indicate significant differences compared to time 0 for each hormone/inhibitor treatment (* *p* < 0.05, ** *p* < 0.01, *** *p* < 0.001; one-way ANOVA with Dunnett correction post hoc). For ABA long-term treatment, asterisks indicate significant differences between treatments (* *p* < 0.05, ** *p* < 0.01), and bars with asterisks indicate significant difference between days of control group; two-way ANOVA with Tukey correction post hoc test was used.

The same pathway also drives the metabolic intermediates to the formation of flavan-3-ols by *FcLAR* and epi-flavan-3-ols by *FcANR* which can be then converted into proanthocyanidins or can provide intermediates for flavonols synthesis by means of *FcFLS*. The level of *FcFLS* transcripts decreases significantly between C1 and C2 stages and remains at a low level during the remaining development stages of *F. chiloensis* fruit (Figure 8A). ABA and Fluridon treatments significantly reduce the expression of *FcFLS* (Figure 8B). During a longer period of time, the transcript accumulation of *FcFLS* displays a tendency to diminish with time in response to ABA (Figure 8C). 

The accumulation of *FcLAR* transcripts showed a peak of expression at the C2 stage (Figure 8D). ABA treatment increments the level of *FcLAR* transcripts after 6 h but recovers to the initial expression level after 24 h; meanwhile, no significant changes were detected by Fluridon treatment (Figure 8E). In response to a long ABA exposure, the expression of *FcLAR* remains unchanged compared to day 0 (Figure 8F). Finally, the level of *FcANR* transcripts decreases significantly between C1 and C2 stages and remains at a low level in the remaining stages (Figure 8G). In response to ABA treatment, *FcANR* transcripts significantly decrease after 6 h and 24 h of treatment; meanwhile, Fluridon does not modify the level of transcripts (Figure 8H). In the longer time, no changes in *FcANR* transcripts were observed in ABA-treated fruit (Figure 8I). In summary, there is a notorious reduction in the expression of *FcFLS* in response to ABA, in addition to a transitory reduction in *FcANR* (6 h, 24 h) and a temporal increment in *FcLAR* (6 h).

### 2.4. Expression Level of FcMYB1

**Figure 9:**
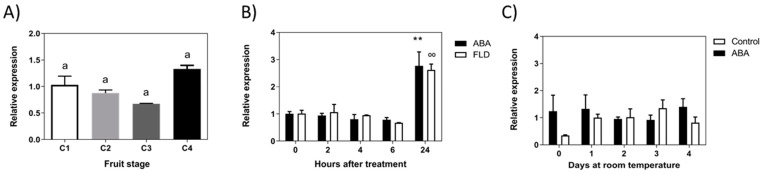
**Expression level of *FcMYB1* TF gene during development of *F. chiloensis* fruit and in response to ABA treatment**. Fruit at C2 stage was immersed in 1 mM ABA or 100 µM Fluridon (short-term treatment) or fruit pedicel was submerged in 1 mM ABA or only buffer (long-term treatment). (**A**) Expression level of *FcMYB1* during development and ripening of the fruit. (**B**) Changes in the expression level of *FcMYB1* in response to ABA and FLD during 24 h of treatment. (**C**) Changes in the expression level of *FcMYB1* in response to ABA during 4 days of treatment. In expression analysis during development progress of *F. chiloensis,* different letters indicate significant differences between fruit stages (one-way ANOVA with Dunnett correction post hoc). In ABA short-term treatments, asterisks or circles indicate significant differences compared to time 0 for each hormone/inhibitor treatment (** *p* < 0.01; one-way ANOVA with Dunnett correction post hoc). For ABA long-term treatment, asterisks indicate significant differences between treatments, different letters indicate significant differences between days of ABA treatment, and bars with asterisks indicate significant difference between days of control group; two-way ANOVA with Tukey correction post hoc test was used.

*MYB1* has been proposed, in addition to other TFs (WD40 and bHLH), as a key regulator of structural genes in the anthocyanins biosynthesis pathway. The accumulation of *FcMYB1* transcripts showed no changes during the development of the fruit (Figure 9A). In response to ABA, the expression of *FcMYB1* remains unchanged but a transitory increase after 24 h of treatment is noticed; Fluridon treatment also increases *FcMYB1* transcripts after 24 h (Figure 9B). In the long-term treatment, non-significant changes were confirmed in ABA-treated fruit (Figure 9C). 

## 3. Discussion

### 3.1. Changes in Fruit Appearance and Transcriptional Changes during Fruit Development

The data provided indicated that *F. chiloensis* develops a pinkish appearance which is due to the production of a reduced level of anthocyanins. According to previous reports, the main anthocyanins produced are pelargonidin-3-glucosides. The level of total anthocyanins in ripe *F. chiloensis* fruit is lower than the level generally quantified in several *F. x ananassa* cultivars. Here, the level of anthocyanins was quantified is 0.45 mg/100 g FW and recent values reported for different *F. x ananassa* cultivars are around 17–22 mg/100 g FW at the ripe stage [18].

The complete transcriptional profile of genes involved in the phenylpropanoid and flavonoid biosynthesis pathways has been previously described during the development and ripening of *F. chiloensis* fruit [10]. In general, the accumulation of transcripts detected here for most of the genes involved in the pathway (Figure 6, Figure 7 and Figure 8) is similar to those reported previously by Salvatierra et al. (2010) [10], except for *FcC4H* and *FcUFGT*. The difference could be explained by the fact that new sets of specific primers were designed for the qPCR analyses of these genes that assured unique PCR products.

As a way to understand and explain the changes in color, metabolites, and the transcription level of genes, correlation analyses were performed (Table 1). During the development of *F. chiloensis* the fruit receptacle develops a pink color appearance, which is accompanied by the increment of anthocyanins in the fruit. The correlation coefficient between color ‘a’ and anthocyanins is 0.87. In parallel with pink color development in *F. chiloensis* fruit, there is a general reduction in the level of phenolics, flavonoids, and antioxidant capacity throughout fruit development (correlation values are 0.95, 0.97, 0.95, and 0.90 for phenolics, flavonoids, FRAP, and DPPH, respectively). The increment in pink color can be endorsed to the increased expression of genes from the phenylpropanoids and flavonoids biosynthesis pathways such as *FcPAL4* (r-value = 0.90), *FcC4H* (r = 0.88), *FcCHS* (r = 0.91), CHI (r = 0.99), DFR1 (r = 0.91), and ANS (r = 0.99). Besides, a negative correlation was determined for genes driven to the synthesis of non-anthocyanin compounds such as FLS (r = −0.90) and ANR (r = −0.87), leading in this way to the synthesis of glycosylated anthocyanins and avoiding the synthesis of flavonols and epi-flavan 3-ols. The reduction in flavonoids content observed during *F. chiloensis* development correlates with the reduction in the expression of FLS (r = 0.89). On the other hand, the fruit antioxidant capacity of the fruit correlates well with the level of phenolics (r = 1 for FRAP, r = 0.98 DPPH) and flavonoids (r = 0.99 for FRAP, r = 0.94 DPPH). Interestingly, *FcLAR* behaves differently, as it displays a unique expression profile, and non-significant correlation values were determined with none of the descriptors.

In general, MYB1 displays a low correlation value with most of the parameters under study; however, a medium correlation value was observed for anthocyanin content (r = 0.66).

### 3.2. ABA Accelerates the Pinkish Color of F. chiloensis Fruit

**Figure 10:**
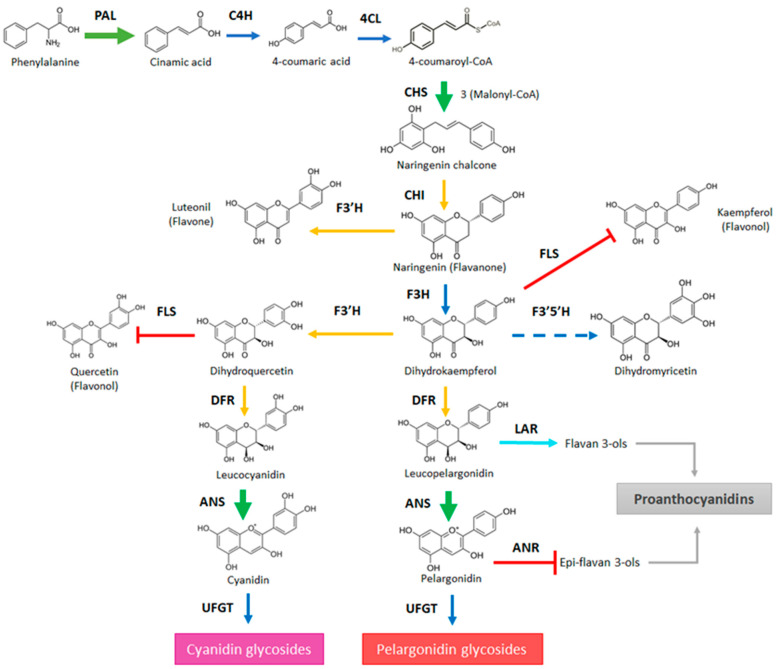
General view of the phenylpropanoid/flavonoid pathway highlighting the expression changes in response to ABA treatment that explains the increment of color in *Fragaria chiloensis* fruit. In the scheme, green arrows indicate the increment in expression promoted by ABA; light blue arrow indicates a transitory increment in the expression; blue arrows indicate no changes in the expression level; yellow arrows denote a transient reduction in the expression followed by the increment in expression; red arrows indicate a decrease in the expression level; dashed blue line indicate that transcripts for the gene were not detected in the fruit. The metabolic pathway has been modified from [19].

The treatment of *F. chiloensis* fruit with ABA accelerates its color development (Figure 3). This was recorded by the increment in the ‘a’ value quantified by the colorimeter and accompanied by the increment in the anthocyanins level. Changes in transcripts were detected in the phenylpropanoids/flavonoids and anthocyanin pathways (Figure 10). ABA induces the expression of *FcPAL2* and *FcPAL4*, the first enzyme committed to the pathway, in addition to the transient induction of *FcCHS* and repression of *FcCHI* and *FcF3′H*. The increment in the expression of *FcCHS* is avoided by the use of Fluridon, suggesting its dependence on ABA. Importantly, the gene coding for *FcANS,* the enzyme directly involved in the synthesis of anthocyanins, responds to ABA with a temporal reduction during the first hours followed by a notorious increment in its transcript level during the following days. Again, the temporal expression changes promoted by ABA in *FcANS* are avoided in Fluridon-treated fruit, confirming its ABA dependence. These changes were also accompanied by the temporary reduction in the expression of *FcDFR1* and *FcUFGT*, a notorious permanent repression of *FcFLS*, and a temporary repression of *FcANR*. These transcriptomic changes explained the increment in anthocyanins induced by ABA, allowing the conversion of Phe into the metabolic intermediates driving the synthesis to anthocyanins, and reducing the conversion of them into glycosylated compounds. In addition, they avoided the conversion of intermediates into other metabolic products of this metabolic pathway such as flavonols or epi-flavan 3-ols; the latter can then be transformed into proanthocyanidins.

Remarkably, in response to ABA, there was an increment in the total content of flavonoids (Figure 4B). The method of AlCl3 employed for flavonoids determination [20] can detect flavonols and flavones in a strong manner; meanwhile, flavanones and isoflavones are detected but with less intensity. The increment in total flavonoids observed in response to ABA treatment after 3–4 days could be explained by the reduction in *FcFLS* expression that provided more intermediates for the route. Some of them can be converted into anthocyanins, meanwhile others into flavones such as luteonil through *FcF3′H* that recovers initial expression level by day 3. As the AlCl3 method is more sensitive for luteolin (flavone) than for naringenin (flavanone), the conversion of naringenin into luteolin could explain the significant increment in total flavonoids observed in ABA-treated fruit. Nevertheless, this should be confirmed through more precise quantification methods, such as HPLC.

### 3.3. Role of TFs in the Regulation of the Phenylpropanoid Pathway

It has been reported that structural genes taking part in the biosynthesis of flavonoid pigments are modulated in their expression by TFs [14]. These TF proteins interact with the promoter regions of structural genes located at different levels within the biosynthetic pathway. The intricate interaction network between structural genes and TFs allows the accumulation of particular metabolites. Three TFs proteins have been reported to be involved in the regulation of structural genes in the anthocyanins biosynthesis pathway: MYB, WD40, and bHLH [21,22,23,24]. In fact, the interaction between these three TFs regulates the appearance of fruit color.

In *F. chiloensis* fruit, the participation of FcMYB1 has been demonstrated in fruit color development. As a negative regulator, the repression of *FcMYB1* through RNAi induces the expression of anthocyanins in the fruit through the transcriptional activation of *FcANS*, but at the same time, the downregulation of *FcLAR* and *FcANR* [14].

In our hands, ABA induces the expression of *FcMYB1* after 24 h; therefore, we cannot conclude if the changes in expression observed in ABA treated fruit are explained by *FcMYB1*. Probably, the other associated TFs may have a more direct relationship with the effects promoted by ABA.

## 4. Materials and Methods

### 4.1. Fruit Material

White Chilean strawberry fruit was harvested at Purén, Chile, and divided into four different stages (C1, C2, C3, C4) as described by Figueroa et al. (2008) [10].

### 4.2. Hormonal Treatments

Fruit at the C2 stage (medium size fruit with green receptacle and red achenes) was submerged into 1 mM abscisic acid (ABA, PhytoTechnology Laboratories, Lenexa, KS, USA) or 100 µM Fluridon (PhytoTechnology Laboratories, Lenexa, KS, USA) following the procedure recently described [17]. Three replicates of 5 fruit each were collected just after treatment and after 6 and 24 h of treatment.

In addition, exposure to ABA for longer periods was also performed. In this case, the pedicel of the fruit was submerged in 1 mM ABA or only buffer [17]. Sampling was performed daily for up to 4 days (3 replicates of 5 fruit) after color measurements. In both types of experiments, the fruit was immediately frozen in pools with the help of liquid nitrogen and stored at −80 °C until use.

### 4.3. Physiological Parameters

Color was measured with the Chroma Meter Model CR-400 (Konica Minolta Inc., Tokyo, Japan) using the CIEL*A*B* scale: a* (range between green and red), b* (range between yellow and blue), and L* (luminosity index) values were recorded. Each fruit was measured twice on opposite faces at the equatorial region. The chroma value (color saturation) was calculated from a* and b* values according to the formulas described by McGuire (1992) [25].

### 4.4. RNA Extraction, cDNA Synthesis and qPCR Analysis

RNA samples were extracted from 100 mg of frozen *F. chiloensis* fruit tissue following the methodology recently described [17]. Three independent RNA extractions were prepared from each sample bulk and used for cDNA synthesis.

For real-time PCR analyses, specific primers for each gene under analysis were obtained from the literature or de novo designed from the 5′-UTR region by Primer3 (http://frodo.wi.mit.edu/primer3) (accessed on 9 September 2019) (Table S1, see Supplementary Materials). The qPCR procedure as described in [17] was carried out. qPCR reactions were carried out in triplicates.

Relative expression data correspond to the means of three biological replicates ± SE normalized against *FcGAPDH* (short-term ABA treatment) or *Fc18S* (long-term ABA treatment), employing the initial time (0 h, 0 day of control group) as a calibrator.

### 4.5. Extraction of Metabolites

Methanolic extracts were prepared from frozen samples. For that, 1 g of sample was homogenized in 5 mL of methanol:HCl (99:1) solution and ground in Ultraturrax (Labsynth, Brazil) for 5 min at 4000 rpm. Extracts were stirred overnight at 4 °C and then centrifuged at 10,000× *g* rpm for 5 min. The supernatant was collected and used for the analysis of several metabolites. Three independent methanolic extracts were prepared from each sample.

### 4.6. Total Phenolics Content

Determinations were performed according to Singleton et al. (1999) [26]. From methanolic extracts, dilutions with 95% methanol were prepared until reaching concentrations of 100 mg mL^−1^. Samples were oxidized with the Folin-Ciocalteu reagent (Sigma-Aldrich, St. Louis, MO, USA) and neutralized with 700 mM sodium carbonate (pH 10) (2 h, room temperature). Absorbance was measured at 765 nm using the Epoch microplate spectrophotometer™ 2 (Agilent Technologies, Santa Clara, CA, USA). A standard curve was prepared with gallic acid (Sigma-Aldrich, St. Louis, MO, USA), and results were expressed as mg of gallic acid equivalents per 100 g of fresh weight (GAE 100 g^−1^ FW).

### 4.7. Flavonoids Content

The colorimetric aluminum chloride method of Chang et al. (2002) [20] was employed with modifications. Diluted methanolic extracts were mixed with 0.1 mL of aluminum chloride (10%), 0.1 mL 1 M potassium acetate, and 4.7 mL of methanol (80%), and incubated for 30 min at room temperature. Absorbance was measured at 425 nm. Quercetin (Sigma-Aldrich, St. Louis, MO, USA) dissolved in ethanol 80% was used for the calibration curve (1–100 μg mL^−1^). Results were expressed as mg of quercetin equivalents per 100 g FW (QE 100 g^−1^ FW).

### 4.8. Anthocyanins Determination

The differential pH method described by Lee et al. (2008) [27] was followed. For that, 200 μL of the extract was mixed with 800 μL of 0.025 M potassium chloride (pH 1.0) or 800 μL of 0.4 M sodium acetate buffer (pH 4.5). After 30 min of incubation at room temperature, the absorbance at 524 and 700 nm was measured. Anthocyanin content was expressed as mg equivalent of cyanidin 3-glucoside (Sigma-Aldrich, St. Louis, MO, USA) per 100 g FW using absorbance values of A = (A524 nm − A700 nm) pH 1.0 − (A524 nm − A700 nm) pH 4.5, considering a molar extinction coefficient of 26,900.

### 4.9. Ferric Reducing Antioxidant Content (FRAP)

FRAP was quantified based on Benzie and Strain (1996) [28]. A ferric complex was prepared by mixing 25 mL of 300 mM sodium acetate (pH 3.6), 2.5 mL of 10 mM TPTZ (2,4,6-Tri(2-pyridyl)-s-triazine) (Sigma-Aldrich, St. Louis, MO, USA) (dissolved in 40 mM HCL) and 2.5 mL of 20 mM FeCl_3_∙6H_2_O. One hundred microliters of methanolic extract was mixed with 3 mL of FRAP reagent and incubated at 37 °C for 4 min; absorbance was measured at 593 nm. Quantification was contrasted with a Trolox standard curve (60 to 300 μM) (Sigma-Aldrich, St. Louis, MO, USA). The results were expressed in µmol Trolox equivalents per 100 g FW.

### 4.10. Total Antioxidant Content (DPPH)

The discoloration method of 1,1-diphenyl-2-picrylhydrazyl (DPPH) proposed by Brand-Williams et al. (1995) [29] was employed. Fifty microliters of each sample extract was diluted in methanol and mixed with 950 μL of DPPH radical reagent (0.06 mM) (Sigma-Aldrich, St. Louis, MO, USA). The mixture was incubated for 30 min at room temperature, and absorbance readings were taken at 517 nm. Methanol was used as a negative control. A calibration curve was prepared using Trolox as the standard (60 to 300 μM). Absorbance was converted to percentage antioxidant activity (AA) using AA% = 100 − ((Abs sample/Abs control) × 100). Results were expressed as μmol Trolox equivalents per 100 g FW.

### 4.11. Correlation Studies

Correlation analyses were performed using R software (1.3.1093) (accessed on 22 November 2021) using ‘ggplot2’ and ‘corrplot’ packages. Pearson’s correlations were obtained (significance *p* < 0.05).

### 4.12. Statistical Analysis

For color changes in response to ABA treatment, a random design with 3 biological replicates of 5 fruit each was employed, using a disappeared *t*-test with Bonferroni correction post hoc. Differences were considered statistically significant when *p* < 0.05 (*).

For gene expression analyses, the strategy reported in [17] was employed.

## 5. Conclusions

The data provided here indicate that ABA promotes important changes in *F. chiloensis* fruit at the C2 stage, such as the advancement in time of firmness reduction (reported elsewhere [17]), color development (Figure 3), and the reduction in total phenolics and antioxidants observed during its normal development (Figure 4). These phenotypic changes are coincident with the increased expression of *FcPAL*, *FcCHS*, and *FcANS* which could explain the advancement of color development due to anthocyanin synthesis (Figure 10). This confirms that ABA has a crucial role in the promotion of ripening-related changes such as color development and the level of antioxidants in this non-climacteric fruit.

## Figures and Tables

**Table 1:** Pearson correlation analysis between color parameters, secondary metabolites, antioxidant capacity, and genes (significant values are shown in bold, *p* < 0.05).

	Color chroma	Color a	Anthocyanins	Phenolics	Antiox (FRAP)	Antiox (DPPH)	Flavonoid	*FcMYB1*	*FcPAL2*	*FcPAL4*	*FcC4H*	*Fc4CL*	*FcCHS*	*FcCHI*	*FcF3’H*	*FcF3*H	*FcDFR1*	*FcANS*	*FcUFGT*	*FcFLS*	*FcLAR*	*FcANR*
Color chroma	1																					
Color a	**−0.98**	1																				
Anthocyanin	−0.82	0.87	1																			
Phenolic comp	**0.99**	**−0.95**	−0.76	1																		
Antiox (FRAP)	**0.99**	**−0.95**	−0.77	1	1																	
Antiox (DPPH)	**0.95**	−0.90	−0.76	**0.98**	**0.98**	1																
Flavonoid	**0.99**	**−0.97**	−0.76	**0.99**	**0.99**	0.94	1															
*FcMYB1*	−0.11	0.23	0.66	−0.02	−0.03	−0.06	−0.01	1														
*FcPAL2*	0.86	−0.88	−0.59	0.82	0.82	0.69	0.90	0.10	1													
*FcPAL4*	−0.85	0.90	0.69	−0.79	−0.79	−0.65	−0.87	0.09	**−0.98**	1												
*FcC4H*	−0.94	0.88	0.59	**−0.97**	**−0.97**	−0.94	**−0.97**	−0.21	−0.84	0.77	1											
*Fc4CL*	0.58	−0.71	−0.69	0.47	0.47	0.32	0.57	−0.46	0.75	−0.86	−0.37	1										
*FcCHS*	**−0.96**	0.91	0.62	**−0.97**	**−0.97**	−0.93	**−0.98**	−0.18	−0.88	0.82	**1.00**	−0.44	1									
*FcCHI*	**−0.98**	**0.99**	0.80	−0.94	−0.94	−0.86	**−0.98**	0.12	−0.94	**0.95**	0.90	−0.72	0.93	1								
*FcF3’H*	−0.74	0.81	0.57	−0.67	−0.67	−0.51	−0.77	0.04	**−0.96**	**0.98**	0.67	−0.89	0.72	0.87	1							
*FcF3H*	−0.80	0.70	0.32	−0.85	−0.85	−0.82	−0.85	−0.50	−0.76	0.63	**0.95**	−0.16	0.94	0.76	0.56	1						
*FcDFR1*	−0.88	0.91	0.63	−0.83	−0.83	−0.70	−0.91	−0.04	**−1.00**	**0.99**	0.84	−0.78	0.88	**0.96**	**0.97**	0.74	1					
*FcANS*	**−1.00**	**0.99**	0.82	**−0.98**	**−0.98**	−0.92	**−0.99**	0.12	−0.90	0.90	0.93	−0.65	**0.95**	**0.99**	0.80	0.78	0.92	1				
*FcUFGT*	0.91	−0.91	−0.60	0.89	0.89	0.78	**0.95**	0.14	**0.99**	**−0.95**	−0.91	0.67	−0.94	**−0.96**	−0.91	−0.84	**−0.99**	−0.94	1			
*FcFLS*	0.86	−0.90	−0.63	0.82	0.81	0.68	0.89	0.02	**1.00**	**−0.99**	−0.82	0.80	−0.86	**−0.95**	**−0.97**	−0.71	**−1.00**	**−0.91**	**0.98**	1		
*FcLAR*	0.15	−0.11	−0.46	0.18	0.19	0.37	0.05	−0.57	−0.36	0.30	−0.03	−0.27	0.01	0.03	0.46	0.13	0.32	−0.08	−0.27	−0.33	1	
*FcANR*	0.83	−0.87	−0.61	0.77	0.77	0.63	0.86	0.01	**0.99**	**−0.99**	−0.77	0.83	−0.82	−0.93	**−0.99**	−0.67	**−0.99**	−0.88	**0.96**	**1.00**	−0.38	1

## Data Availability

All data generated or analyzed during this study are included in this published article.

## Supplementary Materials

The following supporting information can be downloaded at: https://www.mdpi.com/article/10.3390/ijms23073854/s1.
