# Supplementary material for: ABA Speeds Up the Progress of Color in Developing F. chiloensis Fruit through the Activation of PAL, CHS and ANS, Key Genes of the Phenylpropanoid/Flavonoid and Anthocyanin Pathways
Source: Int J Mol Sci. 2022 Mar 31;23(7):3854. doi: 10.3390/ijms23073854 (PMC8998795; doi:10.3390/ijms23073854)
Supplement: Supplementary file 1 [file ijms-23-03854-s001.zip › ijms-1623280-supplementary.pdf]

Supplementary Table S1. Nucleotide sequences of primer pairs employed in this study.

Gene		Sequence (5′ → 3′)	Efficiency (%)	Source
FcMYB1	Fw	GGTTGAGTTGAATCTC	97.3	Synthesis / [11]
	Rv	GCAACTTGAGGATCAGCC		
FcPAL2	Fw	GTGAACCTCTTCCGATATGCTA	96.0	Synthesis
	Rv	CGATTAAAGCTAGGAATCCTCATCA		
FcPAL4	Fw	TGCAATTCCTATAGAGGGTTCTTGA	100.7	Synthesis
	Rv	GGCACAAATGTGTCTTTACCCA		
FcC4H	Fw	CCTCCCCCCTCATAAAACCCACCA	110.0	Synthesis
	Rv	CGATGATTGCGACTATAGCGA		
Fc4CL	Fw	ACTTGGTCAGGGATATGGGATG	108.0	[11]
	Rv	GCACCAGTTTCAGGTCTACG		
FcCHS	Fw	CGTCTCTCGACACTTCTCCG	100.8	Synthesis
	Rv	GGGTACGTGCTCTGGTCAAT		
FcCHI	Fw	TGGTAGGAGGTAGATGGCCGC	99.7	Synthesis
	Rv	GGTGGTAGTTGGGACTTATCGT		
FcF3H	Fw	GGGAGGGGTTTGGTATAGCTT	96.1	Synthesis
	Rv	GGTGTGGGGTGAAGGGGTGGTATAT		
FcF3′H	Fw	CAGAGAGTGGTGCGGAAAAG	97.9	Synthesis
	Rv	CCTTCAACACCATATCGACTCA		
FcDFR1	Fw	TAACTAAGGCATGGGGTTGG	95.9	Synthesis
	Rv	CCTGTGGTAACTCCAGCAGA		
FcANS	Fw	ACCCATAACTATAACTGTTTAAGGGCT	97.9	Synthesis
	Rv	TTGATCTTCGCAGCCAGTGT		
FcUFGT	Fw	ACCTAGGGTAGCAGCGTACA	94.7	Synthesis
	Rv	GTCATGCTCTTGCTCGGCAAC		
FcFLS	Fw	TTATCTTTGGGGTTAGGGCTTGAA	100.0	Synthesis
	Rv	GAGAATGGTGAGGGCGGACA		
FcLAR	Fw	GGTGATGGCACGGTTAAAGC	100.4	[11]
	Rv	CTCCCACAGTGAAGCAAGTCC		
FcANR	Fw	AACTAGAAACCGGTGGTCTTTGT	90.0	Synthesis
	Rv	GCTGAAGTTCTCAGCATGTTATGAT		
Fc18S	Fw	ATTTCGGTCCTATTCTGTTGGC	96.0	[30]
	Rv	GCTTTCGCAGTTGTTCGTCTTT		
FcGAPDH	Fw	GATCTACCTTGGCGAAACCA	96.0	[31]
	Rv	GAGGCGGATCATGTGAACTT		
